# Variation in Structure of a Protein (H2AX) with Knowledge-Based Interactions

**DOI:** 10.1371/journal.pone.0064507

**Published:** 2013-05-31

**Authors:** Miriam Fritsche, Ras B. Pandey, Barry L. Farmer, Dieter W. Heermann

**Affiliations:** 1 Institute for Theoretical Physics, University of Heidelberg, Heidelberg, Germany; 2 Department of Physics and Astronomy, University of Southern Mississippi, Hattiesburg, Mississippi, United States of America; 3 Materials and Manufacturing Directorate, Air Force Research Laboratory, Wright Patterson Air Force Base, Ohio, United States of America; Russian Academy of Sciences, Institute for Biological Instrumentation, Russian Federation

## Abstract

The structure of a protein (H2AX) as a function of temperature is examined by three knowledge-based phenomenological interactions, MJ (Miyazawa and Jernigan), BT (Betancourt and Thirumalai), and BFKV (Bastolla et al.) to identify similarities and differences in results. Data from the BT and BFKV residue-residue interactions verify finding with the MJ interaction, i.e., the radius of gyration (*R_g_*) of H2AX depends non-monotonically on temperature. The increase in *R_g_* is followed by a decay on raising the temperature with a maximum at a characteristic value, *T_c_*, which depends on the knowledge-based contact matrix, *T_cBFKV_* ≤ *T_cMJ_* ≤ *T_cBT_*. The range (*ΔT*) of non-monotonic thermal response and its decay pattern with the temperature are sensitive to interaction. A rather narrow temperature range of *ΔT_MJ_ ≈ 0.015–0.022* with the MJ interaction expands and shifts up to *ΔT_BT_ ≈ 0.018–0.30* at higher temperatures with the BT interaction and shifts down with the BFKV interaction to *ΔT_BFKV_ ≈ 0.011–0.018*. The scaling of the structure factor with the wave vector reveals that the structure of the protein undergoes a transformation from a random coil at high temperature to a globular conformation at low temperatures.

## Introduction

Interactions and temperature are critical in modulating the structure of a protein, a subject of intense interest particularly in computer simulation modeling for decades [1]–[14]. Coarse-graining has become a common practice in modeling of proteins especially in characterizing the interactions among the constituents of proteins and the underlying matrix. Residue-residue interaction based on ensembles of their contact maps (derived from structures of thousands of proteins in the protein data bank) provides a valuable method to analyze the structural response of specific proteins. A number of such knowledge-based contact potentials [1]–[11] have been developed, re-examined and redeveloped using viable approximations to understand the folding dynamics of proteins over the years. Miyazawa and Jernigan (MJ) [2], [3] proposed a knowledge-based contact interaction using an effective medium approach in the spirit of a mean-field approximation after an early proposal by Tanaka and Scheraga [1]. Betancourt and Thirumalai (BT) [7] re-examined the classical MJ contact matrix and the potential matrix by Skolnick et al. [11] and selected a specific solvent reference (Thr) within the Miyazawa and Jernigan scheme [2], [3]. They found [7] that their interaction matrix provides ‘hydrophobicities that are in very good agreement with experiment.’ Bastolla et al. (BFKV) [8] have examined some of these knowledge-based interaction potentials and presented a scheme to guarantee optimal stability for most representative structures. Very recently we have studied the thermal response of the structure of proteins (H2AX) using the classic MJ contact potential. Based on the phenomenological nature of the knowledge-based interactions, it is important to re-analyze the thermal response with additional (presumably better tested and improved) potentials such as BT [7] and BFKV [8]. The main goal is to compare the results of three knowledge-based potentials and identify similarities and differences. Very recently we have carried out a similar analysis [13] on a similar protein (H3.1) of the same histone family to assess the reliability of the coarse-grained representation of the knowledge-based phenomenological interaction. Despite the similarity (histone family and comparable size), the two proteins (H3.1 and H2AX) respond differently to temperature, i.e., globular to random-coil monotonic transition (H3.1) versus nonomonotonic temperature dependence (H2AX) [12]. Therefore, it is important to verify the reliability of the thermal response of H2AX with different knowledge-based potentials.

## Model and Methods

For our ongoing effort, we focus on histone H2AX [12] consisting of 143 residues, which play a critical role in directing the structure of DNA in the nucleosome. In our coarse-grained approach [12], [13], the protein (H2AX) is described by 143 nodes each representing its specific residue, tethered together by fluctuating bonds (with the bond length between consecutive nodes fluctuating between *2* and *√10* in units of lattice constant) on a cubic lattice. Despite the simple matrix grid, the degrees of freedom for each residue and peptide bond are ample, much more than that with the fixed bond length frequently used in lattice simulations [15]. Such a bond-fluctuating mechanism has become a common tool in computer simulation modeling of complex systems as is the case for homopolymers [15], proteins [12], [13], membranes [16], and bio-functionalized nano assemblies [17]. It should be pointed out that our coarse-grained protein with fluctuating (i.e., expanding and contracting) covalent bonds between consecutive residues captures much more details (with many more degrees of freedom) than that of the minimalist HP model used for the sensitivity test by Betancourt and Thirumalai [7]. Each residue in our model interacts with the neighboring residues within a range (*r_c_*) with a generalized Lennard-Jones potential,
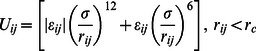
where *r_ij_* is the distance between the residues at site *i* and *j*; *r_c_ = √8* and *σ = 1* in units of lattice constant. The potential strength *ε_ij_* is unique for each interaction pair with appropriate positive (repulsive) and negative (attractive) values used from the knowledge-based contact interactions MJ [2], BT [7], and BFKV [8]. The number of interacting lattice sites (within the range of the interaction) of a residue is relatively large (order of hundred). Because of the efficiency of the approach with the fluctuating covalent bond it is easier to explore the huge structural phase space while incorporating ample degrees of freedom.

Each tethered residue performs its stochastic movements with the Metropolis algorithm briefly described as follows. A residue at a site *i* is selected randomly to move to a neighboring lattice site, *j*. The excluded volume constraints are then checked, including the covalent bond length as a result of the proposed random move. If satisfied, the residue is moved from site *i* to site *j* with the Boltzmann probability *exp(-ΔE_ij_/T)*, where *ΔE_ij_ = E_j_ – E_i_* is the change in energy between its new (*E_j_*) and old (*E_i_*) configuration; *T* is the temperature in reduced units of the Boltzmann constant and the energy (*ε_ij_*). An attempt to move each residue once defines the unit Monte Carlo step (MCS) [15]. We monitor a number of local and global physical quantities during the course of simulation; these quantities include energy of each residue, its mobility, mean square displacement of the center of mass of the protein, radius of gyration and its structure factor. Simulations are performed at each temperature for a sufficiently long time (typically ten million time steps) with many independent samples (typically 150 samples) to estimate the average values of these quantities. We have used a 64*^3^* lattice to generate all the data presented here although different lattice sizes are also used to verify that our findings are independent of the finite size qualitatively.

## Results and Discussion

**Figure 1:**
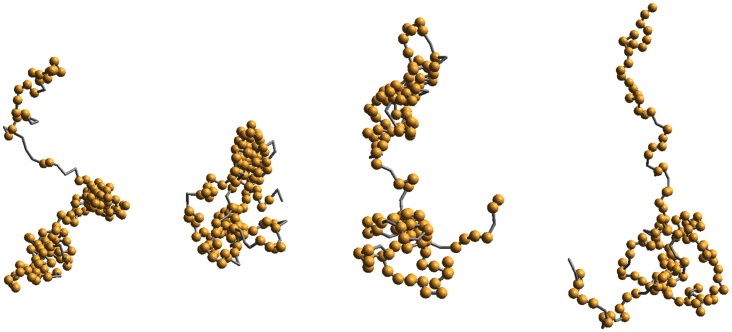
Snapshots of the histone H2AX at *t = 10^7^* time step at the temperatures *T = 0.020, 0.022, 0.025, 0.032* (left to right) using BT potential [7]; residues within the range of interaction are spheres (other than the adjacent).

Figure 1 shows a set of typical snapshots at representative temperatures (spanning low to high) to inspect the variation in size and shape. A snapshot, of course, cannot describe the equilibrium structure as it represents one conformation out of a huge ensemble. Some variations in segmental self-organization and de-segregation resulting in different shapes and sizes can be distinguished visually, however. We see that the aggregation of local aggregates generally appears at low temperatures while the open structures with random coil emerge at higher temperatures. One may guess that the protein can continue to expand on raising the temperature but that is not the case for H2AX. In fact, the radius of gyration of the protein exhibits a non-linear (non-monotonic) response to temperature, a unique characteristic of such a protein (unlike homo-polymers or other proteins) [12] (see below).

**Figure 2:**
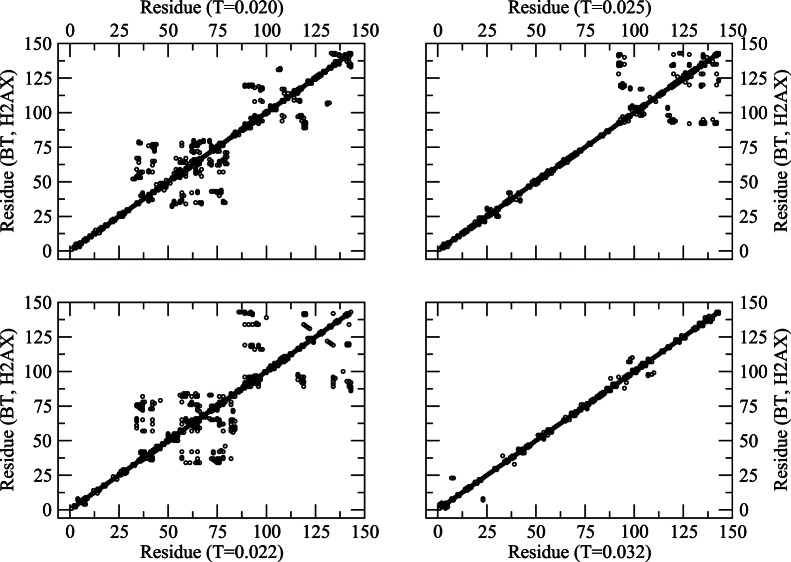
Residue map (neighboring residues along the contour within the range of interaction) of the protein at different temperatures (*T = 0.020–0.032* corresponding to figure 1) with BT potential.

**Figure 3:**
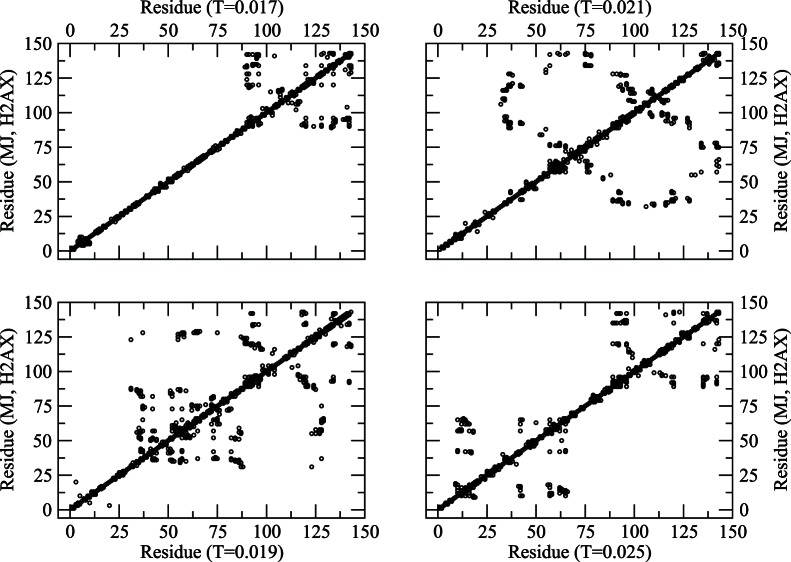
Residue map (neighboring residues along the contour within the range of interaction) of protein at different temperatures (*T = 0.017–0.025*) with the classical MJ potential.

One can also use the residue-residue contact maps to examine the segmental structure of the protein. In figures 2 and 3, we present representative residue maps at representative temperatures (low to high range) with BT and classical MJ potentials, respectively, which provides a first look at the segmental contacts and possible loops. For example, a segmental aggregation appears at *T = 0.020* involving residues at sequence around *30–75*, *90–100*, etc. On raising the temperature to *T = 0.022*, the segmental aggregations re-arrange with somewhat larger loops (sequence *90–143*) while retaining some degree of local self-organization towards a lower sequence (*30–75*). The local assembly (*30–75*) disperses on raising the temperature further to *T = 0.025* while retaining the loops towards the higher end (sequence around *90–140*). Finally, at the relatively high temperature *T = 0.032*, large loops disappear leading to an expanded (random coil) configuration.

**Figure 4:**
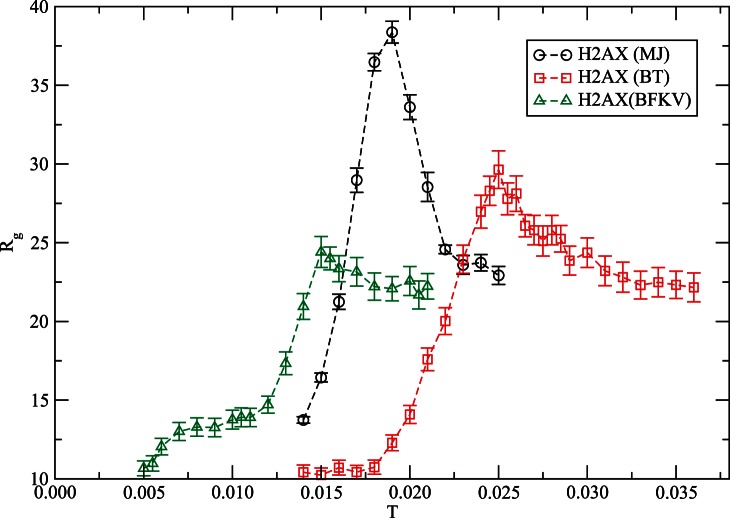
Variation of the average radius of gyration (*R_g_*) of histone H2AX with the temperature using MJ, BT and BFKV potentials. Simulations are performed for *t = 10^7^* MCS time on a *64^3^* lattice with *150* independent samples.

**Figure 5:**
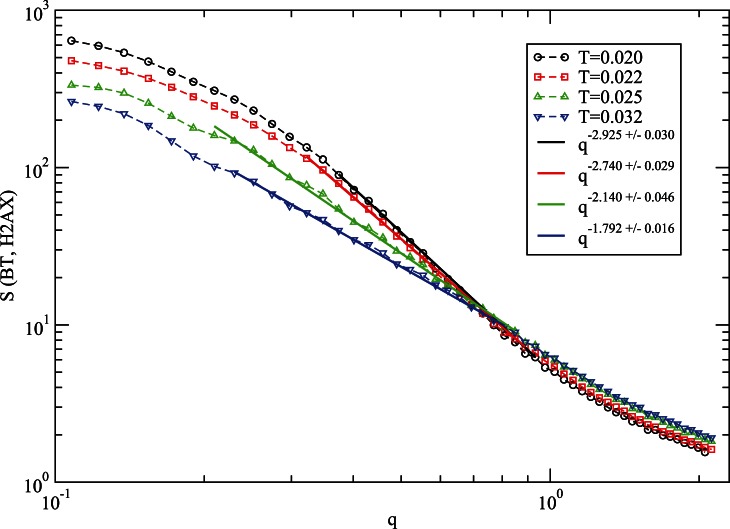
Variation of the structure factor, *S(q)*, of histone H2AX with the wave vector, *q,* with BT potentials from simulations (with *t = 10^7^* MCS time) on a *64^3^* cubic lattice with *150* independent samples.

Qualitatively **s**imulations with the classical MJ potential provide a somewhat similar thermal response (see figure 3), with a different distribution of loops and aggregates. The change in contact map with the temperature in figure 3 does not appear as systematic as that in figure 2 which may be due to differences in interaction potential. The contact map with MJ potential will however converge to that in figure 2 (*T = 0.032*) at a relatively high temperature when the protein conforms to a random coil structure. Note that the contact maps represent a snapshot configuration (from a huge ensemble of conformations) and are presented here to illustrate the differences and similarities among the transient configurations with different potentials. The ensemble averaging over a large number of such configurations provides an estimate of the trends in thermal response of the observable quantities (see below) such as radius of gyration (figure 4) and structure factor (figure 5).

How does the global structure of the protein depend on the choice of the knowledge-based interaction and temperature? As mentioned above, extensive simulations are carried out to evaluate the radius of gyration (*R_g_*) of the protein at a range of temperatures with MJ [2], BT [7] and BFKV [8] potentials. Figure 4 shows the variation of *R_g_* with the temperature (*T*) with three knowledge-based interactions. One can immediately see the differences and similarities in variations of *R_g_* with *T*. Results from all three potentials show non-monotonic response of *R_g_* with *T*. The protein is compact at low temperature (globular conformation, see below); it expands on increasing the temperature until it reaches a maximum value around a characteristic temperature *T_c_*, beyond which it declines. The temperature range over which the non-linear response occurs and the variation pattern (particularly the decay) depends on the potential. The classic MJ potential leads to a sharp thermal response (increase of *R_g_* followed by decay on increasing *T*) in a rather narrow range of temperature *ΔT_MJ_ ≈ 0.015–0.022*. The nonlinear thermal response regime expands *ΔT_BT_ ≈ 0.018–0.030* with the BT potential with a rather broader decay range. With the BT potential, the characteristic temperature (*T_c_*) is also moved upward while the magnitude of *R_g_* is decreased. The change in pattern of the thermal response using BT potential with respect to that of classical MJ potential continues with the BFKV potential where the temperature regime has moved down *ΔT_BFKV_ ≈ 0.011–0.018*.

Which potential is better than others remains elusive due to the lack of experimental data on such a model histone. However, the non-linear thermal response of the structure of H2AX retains the common feature of results. Since the knowledge-based interaction potentials are phenomenological, one should focus on the trend in response properties of the protein rather than on a purely quantitative comparison.

As before [12], [13], we have also studied the structure factor *S(q)* of the protein H2AX as its structure evolves with temperature.
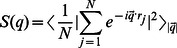
where *r_j_* is the position of each residue and *|q| = 2π/λ* is the wave vector of wavelength, *λ*. From the power-law scaling of the structure factor with the wave vector, *S(q) ∝ q^−1/ν^*, one can estimate the spatial distribution of residues in the protein by analyzing its radius of gyration (*R_g_*). The scaling of the radius of gyration of the protein chain with the number *N* of its nodes (residue), i.e., *R_g_ ∝ N^γ^* provides an insight into the shape of the chain; for axample *γ = ½* represents a random-coil conformation of the protein. Conversely, one can also estimate the effective dimension (*D_e_*) of the residue distributions within the radius (*R_g_*) of the protein, i.e., *N ∝ R_g_^De^, D_e_ = 1/γ*. Estimates of these exponents for shape and mass distribution (*γ, D_e_*) of protein requires evaluation of *R_g_* for a number of different *N*. Unfortunately, we have only a fixed number (*N*) of residues in a protein, therefore, scaling of *R_g_* with *N* is not an option to evaluate the mass distribution (i.e., structure) of the protein. However, we can estimate the exponents of the mass distribution of protein by analyzing the structure factor (follows) over almost all length scales including local segments.

Figure 5 shows the variation *S(q)* with the wave vector *q* with the BT potential. Fitting the data points comparable to size of the protein (*R_g_ ≈ λ*) at appropriate temperatures (see figure 4), we evaluate the effective dimension of the protein. Our data clearly shows a random coil structure (*D_e_≈3*) at the low temperature *T = 0.020* and random coil (*D_e_≈2*) at high temperature *T = 0.032* which is consistent with the results from the classical MJ potential [12]. A closer examination of these data not only exhibits the global conformational response of the protein but also its segmental structure (at higher wavw vector *q*) as well.

### Conclusions

In summary, we have examined the variation in structure of histone H2AX with temperature using three knowledge-based interactions, MJ [2], BT [7], and BFKV [8] applying a coarse-grained Monte Carlo simulation. The variation of the radius of gyration with temperature exhibits a non-monotonic thermal response with all three potentials considered here – a common feature. We confirm the unique characteristics of H2AX [12], i.e., the increase in *R_g_* followed by a decay on raising the temperature with a maximum at a characteristic value *T_c_*. The characteristic temperature (*T_c_*) however depends on the knowledge-based contact matrix, *T_cBFKV_* ≤ *T_cMJ_* ≤ *T_cBT_*; the range over which the non-linear thermal response occurs is also somewhat sensitive to potentials along with the decay pattern of *R_g_* in high temperature regimes. Because of the phenomenological nature of the interaction potential, the qualitative patterns in thermal response should be the main focus rather than the quantitative comparison. Thus, it is important to identify and verify unique characteristics of specific proteins via multiple potentials. We hope that this study will stimulate experimental investigation of H2AX and interpretation of the data based on non-linear thermal response.
